# Alaskana

**Published:** 1893-01

**Authors:** 


ALASKANA, or Alaska in Descriptive and Legendary Poems. By Professor Bushrod W. James, A. M., M. D. Published by Porter & Coates, Philadelphia, 1892. Price, $1.75.

Words seem hardly adequate to describe the contents of this charming volume of trochaic verse by Prof. James, inspired by the scenes he has witnessed in Alaska, and legends he has heard during his long journeys in this far-off land. The rhythm is in conformity with that of Hiawatha, but with the distinction that each chapter is complete in itself. The Legend of Behring's voyage, his discoveries, and his sad fate, is well told. His description of Alaska, physically, is charmingly given. Certainly those who have a love for the beautiful will appreciate this. Two poems are devoted to the flora and birds of Alaska. The manners and customs of the natives are well illustrated. The author being a physician has devoted time and study to the "Medical Men" or "Thamans." Three poems are devoted to them. The poem entitled "Indian River" is especially beautiful. The legends are most poetic and each follows strictly that of the natives. All that can be said to our readers is, read the book! We have not the pen of the poet to illustrate our appreciation of his work, but we congratulate ourselves that Philadelphia has a Longfellow "he the sweetest of all singers," and we hope for other poems from his pen.

The work has some exquisite illustrations which make it even more charming.

Dr. Bushrod W. James hardly needs an introduction to our readers. He is well known as a homœopathic physician practicing in Philadelphia, and one of the most active workers in Hahnemann Medical College of Philadelphia, the Children's Homœopathic Hospital of Philadelphia, and the American Institute of Homœopathy. He is also well known for his long journeys to distant parts of the world. This lovely book is the product of one of the longest of these journeys.

